# Tobacco shred varieties classification using Multi-Scale-X-ResNet network and machine vision

**DOI:** 10.3389/fpls.2022.962664

**Published:** 2022-08-18

**Authors:** Qunfeng Niu, Jiangpeng Liu, Yi Jin, Xia Chen, Wenkui Zhu, Qiang Yuan

**Affiliations:** ^1^School of Electrical Engineering, Henan University of Technology, Zhengzhou, China; ^2^Anyang Cigarette Factory, China Tobacco Henan Industrial Co., Ltd., Anyang, China; ^3^Zhengzhou Tobacco Research Institute of China National Tobacco Corporation (CNTC), Zhengzhou, China

**Keywords:** tobacco shred, image preprocessing, deep learning, classification model, residual neural network, block threshold binarization

## Abstract

The primary task in calculating the tobacco shred blending ratio is identifying the four tobacco shred types: expanded tobacco silk, cut stem, tobacco silk, and reconstituted tobacco shred. The classification precision directly affects the subsequent determination of tobacco shred components. However, the tobacco shred types, especially expanded tobacco silk and tobacco silk, have no apparent differences in macro-scale characteristics. The tobacco shreds have small size and irregular shape characteristics, creating significant challenges in their recognition and classification based on machine vision. This study provides a complete set of solutions aimed at this problem for screening tobacco shred samples, taking images, image preprocessing, establishing datasets, and identifying types. A block threshold binarization method is used for image preprocessing. Parameter setting and method performance are researched to obtain the maximum number of complete samples with acceptable execution time. ResNet50 is used as the primary classification and recognition network structure. By increasing the multi-scale structure and optimizing the number of blocks and loss function, a new tobacco shred image classification method is proposed based on the MS-X-ResNet (Multi-Scale-X-ResNet) network. Specifically, the MS-ResNet network is obtained by fusing the multi-scale Stage 3 low-dimensional and Stage 4 high-dimensional features to reduce the overfitting risk. The number of blocks in Stages 1–4 are adjusted from the original 3:4:6:3 to 3:4:N:3 (A-ResNet) and 3:3:N:3 (B-ResNet) to obtain the X-ResNet network, which improves the model’s classification performance with lower complexity. The focal loss function is selected to reduce the impact of identification difficulty for different sample types on the network and improve its performance. The experimental results show that the final classification accuracy of the network on a tobacco shred dataset is 96.56%. The image recognition of a single tobacco shred requires 103 ms, achieving high classification accuracy and efficiency. The image preprocessing and deep learning algorithms for tobacco shred classification and identification proposed in this study provide a new implementation approach for the actual production and quality detection of tobacco and a new way for online real-time type identification of other agricultural products.

## Introduction

China is a significant producer and consumer of tobacco and related products. In 2019, the total import and export value of China’s tobacco and associated products reached US$3.325 billion. In China’s tobacco and associated products, cigarettes’ export value is 705.4 million US dollars, accounting for 49.68% of tobacco and related products (Beijing, China; National Bureau of Statistics, 2020). The WHO Framework Convention on Tobacco Control (FCTC) implements guidelines for Articles 9 (Regulation of the contents of tobacco products) and 10 (Provisions for tobacco product disclosure) that require manufacturers and importers of tobacco products to disclose to government authorities the composition of tobacco products, including the type of tobacco shred and the blending ratio of each kind of tobacco shred (Acuña A, 2017). Tobacco manufacturers are also required to have the equipment and methods to detect and measure tobacco shred components. The blending amount of the expanded tobacco silk, cut stem, tobacco silk, and reconstituted tobacco shred in cigarettes influences the smoke characteristics, physical indicators, and sensory quality of cigarettes. Therefore, the realization of high-precision and high-efficiency identification of tobacco shred types is of great significance for identifying the authenticity of tobacco products, exploring formula design, and ensuring the quality of the tobacco blending process and the consistency of similar products.

In recent years, deep learning has provided advanced and efficient solutions for image processing tasks, such as image classification (Atila et al., 2021), image segmentation (Kang et al., 2020), and object detection (Janai et al., 2020). Its excellent feature extraction ability greatly reduces the workload of image processing tasks (Shao et al., 2017; Xiao Q. et al., 2019; Afonso et al., 2020). In view of the differences in the application objects, researchers mostly adjust the network structure according to the practical problems (Buiu et al., 2020; Liu et al., 2021; Gu et al., 2021).

In various detection tasks in agriculture, deep learning combined with machine vision has been widely used in plant disease and pest identification, such as wheat blast image classification (Fernández-Campos et al., 2021), rice disease and insect pest image classification (Yang et al., 2021), plant leaf disease classification (Gupta, 2017); plant variety identification, such as vegetable and fruit classification (Zhu et al., 2018; Steinbrener et al., 2019), rapeseed variety classification (Jung et al., 2021); crop quality detection, such as corn seed defect detection (Wang et al., 2022); fruit crop rapid sorting system research, such as citrus online sorting system(Chen et al., 2021), and so on.

With the development and maturity of deep learning technology in agricultural inspection tasks, deep learning research combined with machine vision in the identification and quality inspection of tobacco and its products is also rapidly heating up. Jiao et al. (2022) proposed a tobacco leaf grade recognition method based on a convolutional neural network. This method enhances 1,498 tobacco leaf images of 41 grades to 4,494 for testing, and the final classification accuracy on the test set reaches 95.89%. Lu et al. (2022) proposed a classification method of flue-cured tobacco leaves based on deep learning and multi-scale feature fusion. The process tested a total of 6,068 tobacco leaf images in 7 grades. The final classification accuracy rate is 80.14%. He et al. (2018) proposed an algorithm based on fuzzy pattern recognition. The method classifies the tobacco leaf samples by extracting the appearance features of tobacco leaves. The final accuracy on the training and test sets is 85.81 and 80.23%, respectively. Lu et al. (2021) proposed a tobacco leaf classification method combining a convolutional neural network and a double-branch integral. The technique selected 2,791 flue-cured tobacco leaves of 8 different grades as research samples, and the final tobacco leaf classification accuracy was 91.30%.

There are many related studies on tobacco leaf grading, and they are relatively mature. However, due to the difficulty of obtaining tobacco shred samples, small size, and slight morphological differences, the research on image classification is still lacking. The identification methods of tobacco shred types mainly include manual sorting, near-infrared spectroscopy, and computer vision analysis. The manual sorting method is that experienced workers identify and then complete the sorting work manually. The efficiency of this method is low and the classification accuracy fluctuates significantly due to the influence of artificial subjective experience. The quantification and detection accuracy of indicators cannot be guaranteed. Near-infrared spectroscopy (NIR) detects the spectral information of tobacco shreds to determine the spectral difference and analyze the type of tobacco shred. Since the raw material of reconstituted tobacco shred contains fine tobacco slag, tobacco stems, etc., its spectral information is less different from other types, and the identification error is significant (Jennife, 1999; Liu et al., 2006; Hu et al., 2010; Zhang et al., 2020).

The recognition method based on machine vision completes the type of tobacco shred by analyzing the image features. Dong et al. (2015, 2016a,2016b) proposed a patent for an identification method for establishing a corresponding tobacco shred feature database for different tobacco shred types. This method shows a feature database by extracting the RGB and HSV color space pixel variance values and texture feature values of contrast, entropy, and correlation in the tobacco shred image. Finally, it determines the tobacco shred type based on the correlation threshold of the feature. Gao et al. (2017) proposed a method for identifying the material composition of the tobacco shred based on the LeNet-5 network. This method proves that the macroscopic structure of the tobacco shred is different by analyzing the visible characteristics such as duty cycle, perimeter ratio, and uniformity of tobacco shred. The author cropped the four types of tobacco shred images to a large size to obtain a small picture of 52 × 52 pixels. There are 100 initial tobacco shred images, and 29,208 tobacco shred images are obtained after cropping. The model is trained and tested on the tobacco shred dataset, and the recognition accuracy rates on the training and test samples are 100 and 84.95%, respectively. Zhong et al. (2021) proposed a method for identifying tobacco shred types based on residual neural networks and transfer learning simultaneously. The original dataset used by the author contains a total of 400 images of expanded tobacco silk, cut stem, tobacco silk, and reconstituted tobacco shred. The accuracy rate of the test sample is 97.62%. After data enhancement of the initial dataset, there are 7,832 images with 4 types, and the recognition accuracy of test samples is 98.05%.

In a previously published research, the number of samples in the original tobacco shred dataset was small (100 by Gao et al., 2017 and 400 by Zhong et al., 2021). The high recognition accuracy obtained in these research works is because the sample directly occupies the entire shooting field of view when shooting, and an image with more uniform brightness and more obvious sample characteristics is obtained. In addition, more image samples are obtained by augmenting the original dataset. This method is challenging to apply to composition determination in the field because it cannot place more tobacco shred samples in the shooting field of view and can only be detected by a single piece. The change in shooting field size must be accompanied by the scaling of the tobacco shred image. The difference in features, such as size and brightness, poses a more significant challenge to the model’s generalizability. The original dataset of this method also has fewer samples, and the model has a greater risk of overfitting and may have specificity.

This study mainly aims at the online real-time identification and classification of tobacco shred types and their actual production use in the field. It proposes an overall image processing and classification scheme that realizes the efficient and accurate identification of different tobacco shred types. The main contributions of this study are as follows:

1. A block threshold binarization method for the tobacco shred image is designed. The tobacco shred image is segmented through contour extracting and region of interest (ROI) area cropping. The complete tobacco shred image is then obtained by expanding the ROI area. A dataset containing 8,202 original tobacco shred images is established, effectively avoiding overfitting and specificity.
2. The ResNet50 network is selected as the prominent network architecture and the MS-X-ResNet network is constructed. The constructed network achieves an accuracy of 96.65% on the tobacco shred dataset, outperforming other similar deep learning methods.
3. The focal loss function is introduced to alleviate the influence of different degrees of tobacco shred identification difficulty on the model, effectively improving its accuracy and stability.

## Materials and methods

**FIGURE 1:**
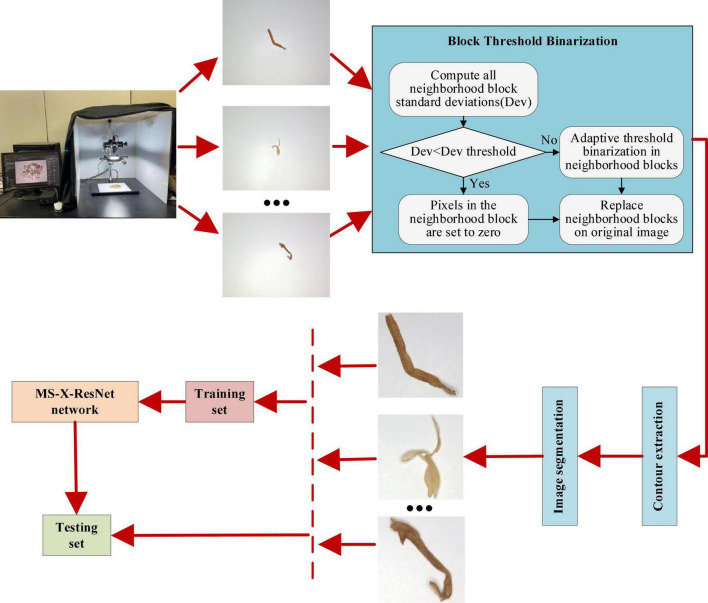
Research flow chart.

This study performs image acquisition, processing, segmentation, dataset establishment, and model building for four tobacco shred types. This section explains all materials and methods in detail, and the research flow chart is shown in Figure 1.

### Materials

**FIGURE 2:**
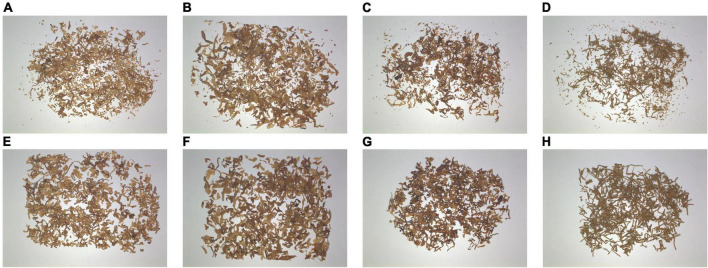
The original images are **(A)** expanded tobacco silk, **(B)** cut stem, **(C)** tobacco silk, and **(D)** reconstituted tobacco shred. Images of tobacco shred after sifting are **(E)** expanded tobacco silk, **(F)** cut stem, **(G)** tobacco silk, and **(H)** reconstituted tobacco shred.

Each cigarette contains four tobacco shred types: expanded tobacco silk, cut stem, tobacco silk, and reconstituted tobacco shred. This study’s samples containing the four tobacco shred types came from the Zhengzhou Tobacco Research Institute of the China National Tobacco Corporation. Figures 2A–D are images of the four unscreened tobacco shred types. The JJSY30x10 circular inspection flat screen produced by Shanghai Jiading Cereals and Oils Instrument Co., Ltd. is used to filter the tobacco shred residues. The sieve surface is 20 mesh (0.9 mm), and a flat sieve is used for 10 s each time. Figures 2E–H shows the tobacco shred images after sifting the residues. Through screening, 2,200 pieces of each of the four types of tobacco shred were obtained, resulting in a total of 8,800 samples.

### Image acquisition

**FIGURE 3:**
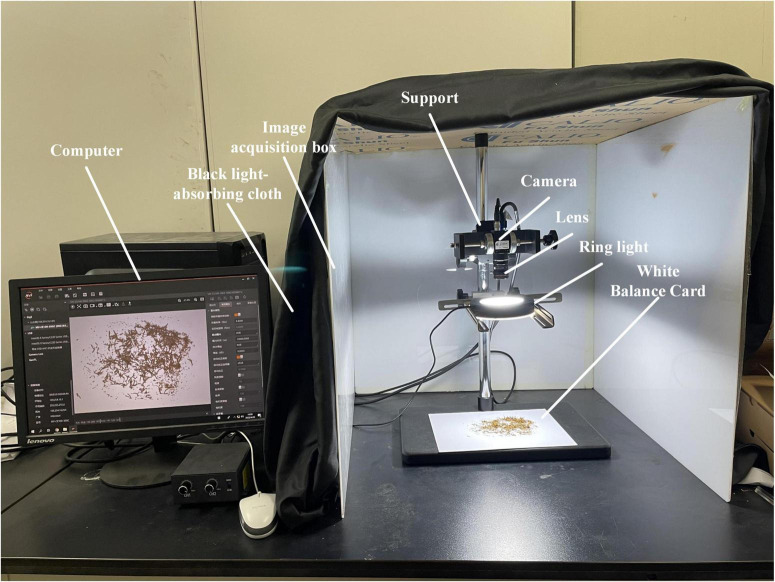
Image acquisition platform.

An image acquisition darkroom with a ring light source was designed to obtain higher quality tobacco shred images. Figure 3 is a physical map of the image acquisition system. The housing size of the image acquisition system is 60 cm × 60 cm × 60 cm, and a black light-absorbing cloth is used outside to prevent interference from external light sources. The camera and the light source are fixed on the support bracket. A Hikvision MV-CE100-30GC 10-megapixel color industrial camera is equipped with a Hikvision MVL-HF1224M-10MP 12 mm focal length industrial lens. The background for the tobacco shot was a standard white balance card to ensure quality.

The official MVS software provided by Hikvision was used for image acquisition. The exposure time was 1/100 s, the sRGB model was selected for gamma correction, and the automatic white balance was turned on. A Huakang Technology 120-80-25 industrial ring angle light source equipped with a diffuser plate to diffuse the light evenly was used to ensure the light source uniformity. The front of the platform is open for easy insertion and removal of tobacco shred samples. The model image is transmitted to a computer *via* an optical fiber. Images were collected for the four shred types with 2,200 pieces each, a total of 8,800 pieces, and the size of each image was 2,788 × 2,238 pixels. Unqualified tobacco shred images, such as blurry and incomplete backgrounds, were manually screened out, and 8,202 tobacco shred images were obtained.

### Image preprocessing

This research on tobacco shred type identification aims to meet the needs of subsequent online tobacco shred component detection in the field. For the component detection of tobacco shreds, it is necessary to simultaneously identify various types of tobacco shreds spread on-site and to calculate and obtain the component ratio. Therefore, our shooting field must be much larger than a single tobacco shred. Then, all shooting platform parameters need not be changed, and the current recognition model and dataset can be directly applied in the subsequent on-site component detection.

The sizes of the original tobacco shred images are 2,788 × 2,238 pixels. The overall image size in the original image is large, and the area occupied by the tobacco shred is small. It is necessary to perform image processing on the original image to facilitate system accuracy and efficient feature recognition to reduce irrelevant information, accelerate model convergence, and improve classification accuracy. The image preprocessing process is as follows: (1) grayscale image; (2) block threshold binarization; (3) obtain the tobacco shred outline; and (4) crop the ROI area.

#### Block threshold binarization: Comparison with other binarization methods

**FIGURE 4:**
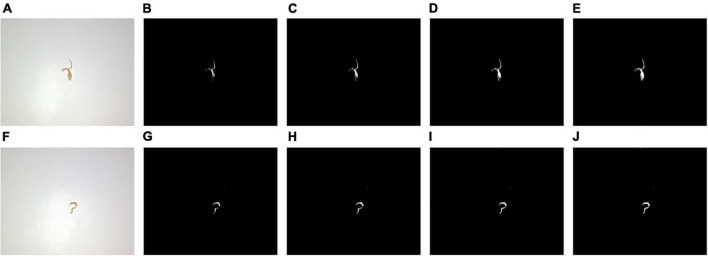
Results of simple threshold binarization under different thresholds **(A)** original image 1, **(B)** threshold 165, **(C)** threshold 170, **(D)** threshold 175, **(E)** threshold 180, **(F)** original image 2, **(G)** threshold 165, **(H)** threshold 170, **(I)** threshold 175, and **(J)** threshold 180.

First, a simple threshold binarization was tested using a fixed threshold to binarize the grayscale image directly. The binarization results using four different thresholds are shown in Figure 4, which shows that different tobacco shred images have different threshold requirements. In Figure 4A, when the threshold is 175 and 180, and in Figure 4F when the threshold is 180, an unbroken binarized tobacco shred image can be obtained without splitting into two contours after processing. There are differences in tobacco shred thickness, light transmittance, and color depth, so different thresholds must be used for other tobacco shreds.

The total performance of the simple threshold binarizations at 165, 170, 175, and 180 thresholds is shown in Table 1. Table 1 shows that different thresholds have almost no effect on the tobacco shred processing (execution) time. The best performing threshold is 175. Among the 8,202 samples, 7,948 binarized tobacco shred images are complete. The proportion of complete samples to the total samples is 96.90% and requires 203.96 s.

**TABLE 1:** Simple threshold binarization performance evaluation table.

Binarization method	Threshold	Complete samples/total samples	The proportion of complete samples (%)	Execution time(s)
Simple threshold	165	7,697/8,202	93.84	203.88
	170	7,876/8,202	96.03	205.08
	175	7,948/8,202	96.90	203.96
	180	7,833/8,202	95.50	204.86

**FIGURE 5:**
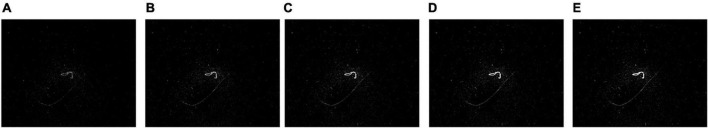
Results of adaptive threshold binarization under different neighborhood block sizes. **(A)** 7 × 7, **(B)** 13 × 13, **(C)** 19 × 19, **(D)** 25 × 25, and **(E)** 31 × 31.

Second, the adaptive threshold binarization is tested. This method sets the threshold according to the local image characteristics and can alleviate the problem of uneven image brightness to a certain extent. The adaptive threshold binarization results using five different neighborhood block sizes are shown in Figure 5. As seen in Figure 5, the neighborhood block size affects the contour’s details. A larger neighborhood block results in a more obvious contour. The interference also increases, and reducing the size of the neighborhood block can filter out more noise.

The performance of the adaptive threshold with the mean and Gaussian weighting methods is shown in Table 2. Table 2 shows that both adaptation methods are time-consuming when the neighborhood block sizes are 7 × 7, 13 × 13, 19 × 19, 25 × 25, and 31 × 31. Gaussian weighting is more time-consuming than the mean method, but performance is also significantly improved. When the size of the neighborhood block is 7 × 7 with the Gaussian weighting method, 7,811 of the 8,202 samples have complete binarized tobacco shred images, and the proportion of complete samples is 95.23%. However, the execution time reaches 9,411.28 s, which is impractical.

**TABLE 2:** Adaptive threshold binarization performance evaluation table.

Binarization method	Adaptive method	Block size	Complete samples/total samples	The proportion of complete samples (%)	Execution time(s)
Adaptive threshold	Mean	7 × 7	6,685/8,202	81.50	6,566.98
		13 × 13	6,321/8,202	77.07	5,249.03
		19 × 19	5,503/8,202	67.09	5,532.24
		25 × 25	5,091/8,202	62.07	6,022.61
		31 × 31	4,986/8,202	60.79	6,139.02
	Gaussian	7 × 7	7,811/8,202	95.23	9,411.28
		13 × 13	6,717/8,202	81.89	6,344.37
		19 × 19	6,583/8,202	80.26	6,065.46
		25 × 25	6,364/8,202	77.59	6,153.02
		31 × 31	6,085/8,202	74.19	5,853.46

**FIGURE 6:**
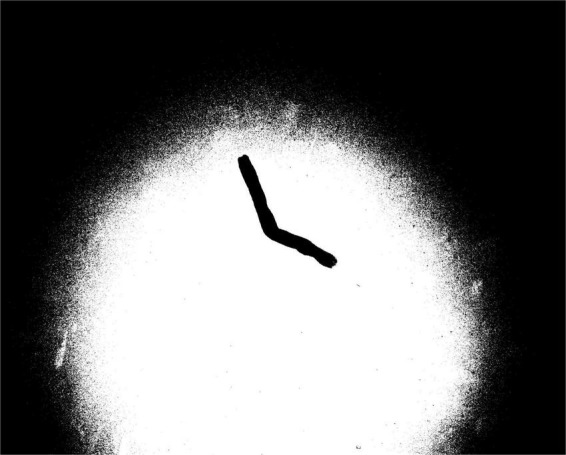
Result of Otsu’s threshold binarization.

Third, Otsu’s threshold binarization is tested. Otsu’s threshold binarization finds a value between the double peaks of the grayscale histogram as a threshold. Since the light source coverage on the camera’s field of view is not entirely uniform, the use of Otsu’s threshold causes an aperture shadow, which generates more spots and increases the calculation amount for subsequent contour screening. Otsu’s threshold binarization effect is shown in Figure 6. The performance of Otsu’s threshold is shown in Supplementary Table 1. This method is time-consuming and has low precision, making it unsuitable for the binarization of tobacco shred images.

The performance of the three threshold binarization methods has been comprehensively analyzed. The simple threshold processing speed is fast, but there are still many cases of incomplete tobacco images. The adaptive threshold method takes too long to be practical. Otsu’s threshold causes many useless contours in the final binarized image, which increases computational and time costs for subsequent contour screening because the bimodal feature of the grayscale tobacco shred image is not apparent. The above three binarization methods are unsuitable for tobacco shred images. Starting from the processing ideas of the adaptive threshold and Otsu’s threshold, this study designs a block threshold binarization method that is applied to tobacco shred images.

The block threshold binarization process is as follows: (1) divide the original image according to the size of the neighborhood block and calculate the standard deviation (SD) of the pixels in the neighborhood block; (2) compare the SD of the neighborhood block with the SD threshold size. When the SD in the tobacco shred block is less than the SD threshold, set all pixel values in the neighborhood block to zero, filter the useless blocks, and reduce the computational cost of subsequent contour screening; otherwise, use Otsu’s threshold to binarize the block; (3) complete the binarization of the original image by traversing all neighborhood blocks.

**FIGURE 7:**
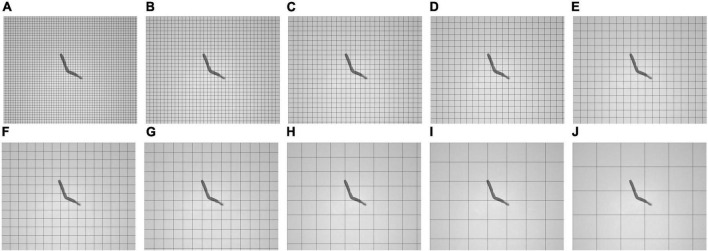
Schematic diagram of image segmentation under different neighborhood block sizes. **(A)** 50 × 50, **(B)** 75 × 75, **(C)** 100 × 100, **(D)** 125 × 125, **(E)** 150 × 150, **(F)** 175 × 175, **(G)** 200 × 200, **(H)** 300 × 300, **(I)** 400 × 400, and **(J)** 500 × 500.

This study explores the neighborhood block size and the SD threshold of the neighborhood block. Figures 7A–J shows tobacco shred images for ten neighborhood block sizes between 50 × 50 and 500 × 500.

Figure 7 shows that a smaller neighborhood block size implies more neighborhood blocks into which the sample is divided. When the neighborhood block sizes are 50 × 50 and 500 × 500, the numbers of neighborhood blocks are 2,520 and 30 blocks, respectively, a factor of 84. From this, it can be concluded that the smaller the neighborhood block size, the greater the number of neighborhood blocks to be processed, and the greater the amount of computation required.

**FIGURE 8:**
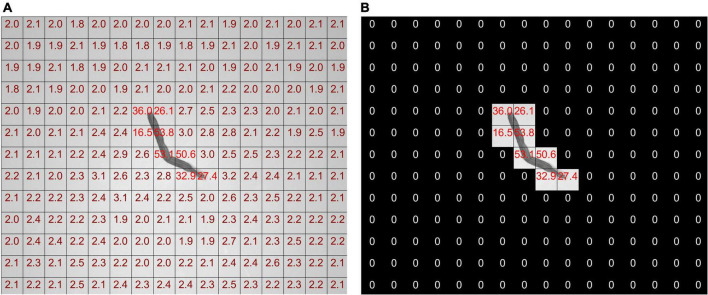
Annotation map of the standard deviation of neighborhood blocks before and after processing **(A)** before, **(B)** after.

The SD threshold is used to process the neighborhood block. The SDs of a tobacco shred image neighborhood block before and after processing are shown in Figure 8.

As shown in Figure 8A, the pixel SD in each neighborhood block is not zero before the tobacco shred image processing. The SD of the neighborhood block containing the tobacco shred is nearly ten times larger than without the tobacco shred. The SD threshold of the neighborhood block can effectively eliminate the background neighborhood blocks that do not contain the tobacco shred image, and the pixel values of the neighborhood blocks to be eliminated are set to zero.

The following discusses finding a neighborhood block threshold suitable to all tobacco shred images and the neighborhood block size with the best performance. As shown in Figure 8A, among the 208 neighborhood blocks, only 8 of them contain parts of the tobacco shred. The SDs of the neighborhood block containing the tobacco shred and the background block are quite different. Therefore, this study uses a statistical method to determine the neighborhood block’s threshold. The neighborhood block’s size is determined according to the complete proportion of the samples and the SD threshold is adjusted.

The steps for determining the threshold of the neighborhood block are as follows: (1) obtain the SD of all neighborhood blocks of the 8,202 tobacco shred samples; (2) draw the SD as a histogram with kernel density. The abscissa of the histogram is the SD, and the ordinate is the number of neighborhood blocks; (3) find the abscissa of the first trough in the kernel density curve and round it down as the neighborhood block threshold.

The principle of the above method is that the number of background neighborhood blocks is very different from the target neighborhood blocks containing tobacco shreds, and the position of the first trough of the kernel density curve is the boundary between the number of target blocks and the standard blocks. The first peak and valley are selected to minimize the errors caused by the following two situations: (1) an image with shredded tobacco shred residues appearing in the background block; (2) an image containing part of a tobacco shred is divided into background blocks. Both cases increase the SD of the background neighborhood blocks. Using subsequent peaks and valleys increases the probability of removing background blocks. It is unreasonable to directly remove the neighborhood blocks in case 2, which would affect the integrity of the tobacco shred image.

**FIGURE 9:**
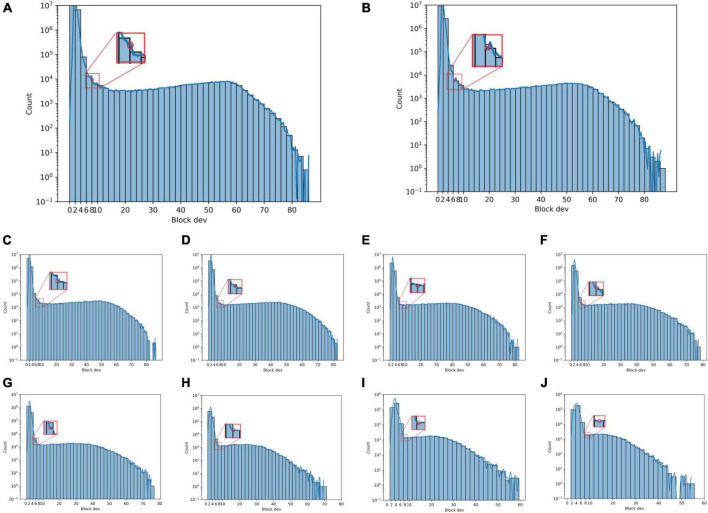
Histogram of neighborhood block standard deviation under different neighborhood block sizes. **(A)** 50 × 50, **(B)** 75 × 75, **(C)** 100 × 100, **(D)** 125 × 125, **(E)** 150 × 150, **(F)** 175 × 175, **(G)** 200 × 200, **(H)** 300 × 300, **(I)** 400 × 400, and **(J)** 500 × 500.

Figures 9A–J show histograms of the SD of the neighborhood blocks for different neighborhood block sizes. The abscissa is the neighborhood block SD, and the ordinate is the logarithm of the number of neighborhood blocks. The SD thresholds for different neighborhood block sizes are obtained from Figure 9 according to the steps for determining the SD threshold.

**FIGURE 10:**
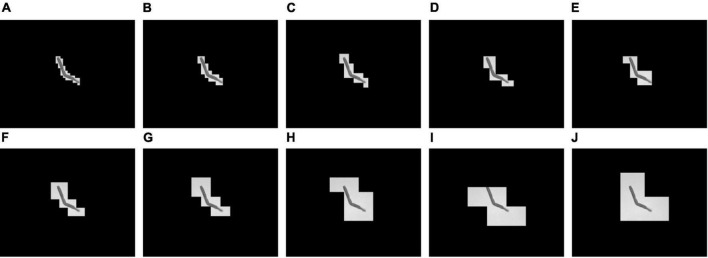
Result of block threshold binarization to remove background under different neighborhood block sizes. **(A)** 50 × 50, **(B)** 75 × 75, **(C)** 100 × 100, **(D)** 125 × 125, **(E)** 150 × 150, **(F)** 175 × 175, **(G)** 200 × 200, **(H)** 300 × 300, **(I)** 400 × 400, and **(J)** 500 × 500.

Figures 10A–J are the tobacco shred images after removing the background using the corresponding SD threshold. As shown in Figure 10, the method for confirming the SD threshold using the statistical neighborhood block SD is effective. However, when the size of the neighborhood block is 400 × 400 pixels, the tobacco shred binarized image is incomplete, indicating that choosing a larger size for the neighborhood block may cause the image to be incomplete. But choosing a smaller size for the neighborhood block increases the computational load, so a tradeoff must be made between neighborhood block size and processing time.

The block threshold’s binarization performance indicators are shown in Table 3. As seen from Table 3, when the neighborhood blocks range from 50 × 50 to 125 × 125, the block threshold performance is good, and the proportion of complete samples is over 98%. The binary value optimization performance is the best when the neighborhood block size is 75 × 75, and the fraction of complete samples is 99.67% but requires 472.74 s. When the neighborhood block size ranges from 175 × 175 to 500 × 500, the proportion of complete samples and the performance decreases as the neighborhood block size increases. Furthermore, when the neighborhood block is 125 × 125 pixels, the SD threshold is reduced from 7 to 6 to retain more original information about the neighborhood block, and the proportion of complete samples reaches 99.29% in 320.01 s. Therefore, this study uses a neighborhood block size of 125 × 125, and a SD threshold of 6 is adopted as the binarization parameter of the block threshold.

**TABLE 3:** Block threshold binarization performance evaluation table.

Block size	Standard deviation threshold	Complete samples/total samples	The proportion of complete samples (%)	Execution time(s)
50 × 50	8	8,157/8,202	99.45	724.25
75 × 75	6	8,175/8,202	99.67	472.74
100 × 100	8	8,044/8,202	98.07	416.57
125 × 125	7	8,085/8,202	98.57	328.92
125 × 125	6	8,144/8,202	99.29	320.01
150 × 150	8	7,824/8,202	95.39	289.94
175 × 175	6	8,044/8,202	98.07	265.87
200 × 200	5	7,951/8,202	96.94	276.52
300 × 300	7	7,569/8,202	92.28	253.85
400 × 400	7	7,362/8,202	89.76	281.64
500 × 500	8	6,841/8,202	83.41	277.85

The performance of the four threshold binarization methods is shown in Table 4. Overall, the block threshold binarization proposed in this study retains more contour information during binarization and has a low execution time, resulting in the best overall performance.

**TABLE 4:** Performance evaluation table of different threshold methods.

Binarization method	Complete samples/total samples	The proportion of complete samples (%)	Execution time(s)
Simple threshold	7,948/8,202	96.90	203.96
Adaptive threshold	7,811/8,202	95.23	9,411.28
Otsu’s threshold	6,896/8,202	84.08	1,544.65
Block threshold binarization	8,144/8,202	99.29	320.01

#### Tobacco shred image segmentation

After the block threshold binarization process, residue contours may still be near the tobacco shred, so we continue to perform contour screening to obtain accurate tobacco shred images. Furthermore, because the same input image length and width are needed for the classification network, this study adjusts the ROI area so that the length and width of the cropped image are the same to prevent the scaling operation from distorting the tobacco shred image proportions.

**FIGURE 11:**
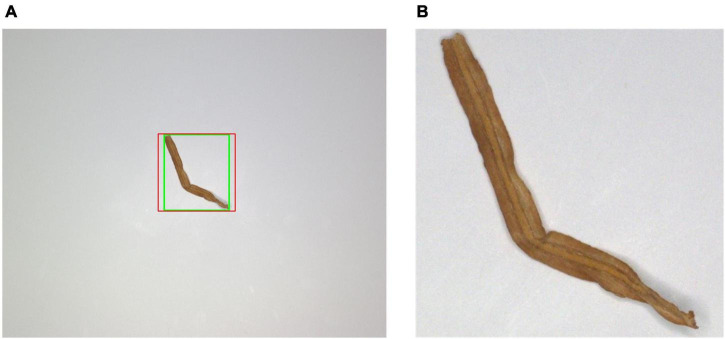
Result of tobacco shred image segmentation **(A)** cropped ROI outline **(B)** segmented tobacco shred image through ROI.

Figure 11 shows some tobacco shred image segmentation results. The green frame is the original ROI area and the red box is the adjusted ROI area in Figure 11A. The segmented tobacco shred image in the ROI is shown in Figure 11B.

### Model construction

#### Dataset construction

Of the 8,202 image samples, 5,741 images were randomly selected as the training set, and the remaining 2,461 images were used as the test set (a 7:3 ratio). The quantity information for each tobacco shred type is shown in Table 5.

**TABLE 5:** Details of tobacco shred dataset.

Name	Expanded tobacco silk	Cut stem	Tobacco silk	Reconstituted tobacco shred	Total
Training set	1,417	1,426	1,413	1,485	5,741
Testing set	606	611	605	639	2,461
Total	2,023	2,037	2,018	2,214	8,202

#### Multi-Scale-X-ResNet model construction

The ResNet50 network is adopted as the primary structure of the neural network. ResNet50 consists of 50 layers of networks, which can be divided into six stages: Stem, Stage 1–Stage 4, and Head. The model’s input is a 224 × 224 pixel tobacco shred image and the output size of Stage 4 is 7 × 7 × 2,048. The tobacco shred image classification is completed through the fully connected (FC) layer, and the model output is the tobacco shred type. The characteristic differences between tobacco shred types are slight, and using 7 × 7 convolution results is less effective for identifying tobacco shreds with smaller sizes.

Furthermore, since the expanded tobacco silk is made from tobacco silk through an expansion process, the proportion of expansion varies. It is difficult to distinguish between tobacco silk and expanded tobacco silk. The shallow feature map has a smaller receptive field and more details of small objects but lacks rich semantic information. The deep feature map has a more receptive lot and rich semantic information but contains less small object information. Therefore, using the multi-scale structure for feature fusion can solve the problem of feature loss to a certain extent. According to the shallow and deep network characteristics, the output results of Stage 3 and Stage 4 of the ResNet50 network are passed through the AvgPOOL layer and the Flatten layer, and the Concat layer is then used for feature splicing the output. The output is passed through the FC layer to obtain the final classification result. Tobacco shreds with small-sized features can be effectively extracted using the multi-scale structure, and the model recognition accuracy can be improved. After adding the multi-scale structure, the resulting network is named MS-ResNet.

Each stage of a ResNet network consists of a sequence of *d* blocks. The numbers of blocks in Stage 1–Stage 4 of ResNet50 and ResNet101 are (3, 4, 6, 3) and (3, 4, 23, 3), respectively. The two networks only have different numbers of blocks for Stage 3. The A-ResNet network is also obtained by changing the number of blocks in Stage 3. In addition, to reduce the complexity of the A-ResNet network and retain more shallow feature information, the number of blocks in Stage 2 of the A-ResNet network is changed to 3 to obtain the B-ResNet network. A-ResNet and B-ResNet are collectively referred to as the X-ResNet network. The numbers of blocks for the different ResNet networks are provided in Supplementary Table 2.

**FIGURE 12:**
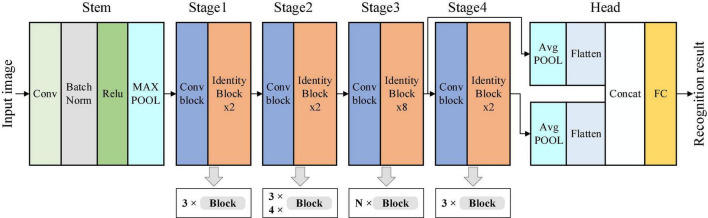
Multi-Scale-X-ResNet network structure.

The MS-X-ResNet network structure proposed in this study is shown in Figure 12. The MS-ResNet network is obtained by fusing the multi-scale features of the ResNet network. The numbers of blocks of Stage 1–Stage 4 are adjusted from the original 3:4:6:3 to 3:4:N:3 (A-ResNet) and 3:3:N:3 (B-ResNet) to obtain the X-ResNet network. The MS-X-ResNet network is obtained by combining the MS-ResNet and X-ResNet networks.

**FIGURE 13:**
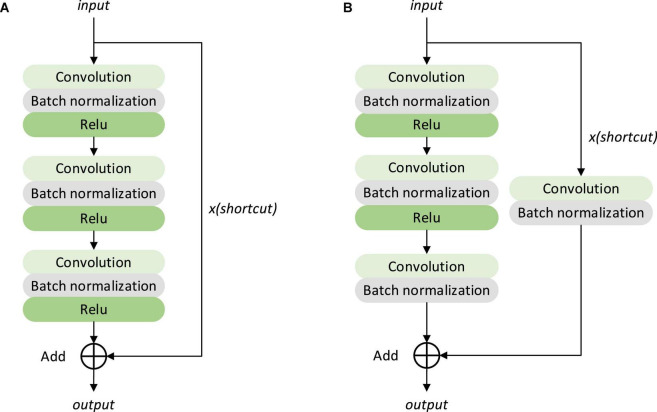
Structure of identity mapping module and convolution module in a residual neural network **(A)** identity block, **(B)** Conv block.

The identity and Conv block structures in Figure 12 are shown in Figure 13.

### Loss function

#### Focal loss

The traditional loss function uses the cross-entropy function, which describes the distance between the actual and expected output probability distributions. The smaller the value of the cross-entropy, the more effective the learning in the model training process (Bishop and Nasrabadi, 2006; Xiao Z. et al., 2019).

Table 6 shows shape images of the four tobacco shred types. As seen from Table 6, the same tobacco shred type can have many different shapes. The difference between tobacco shred images is slight and identification is difficult. However, the traditional cross-entropy as a loss function ignores the identification difficulty of different types of samples. It only focuses on the accuracy of the correct label. In this study, the focal loss function (Lin et al., 2017) is selected to reduce the impact of sample identification difficulty on the network and improve its performance.

**TABLE 6:** Tobacco shred shape image table.

Name	Shape of most samples		Shape of a little samples			
Expanded tobacco silk	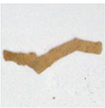	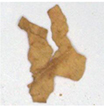	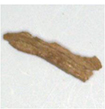	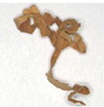	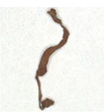	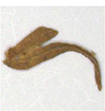
Cut stem	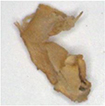	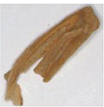	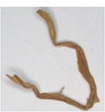	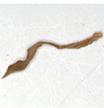	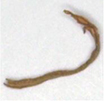	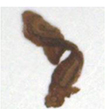
Tobacco silk	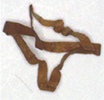	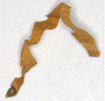	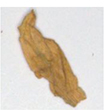	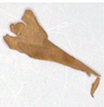	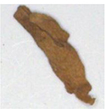	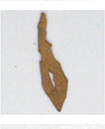
Reconstituted tobacco shred	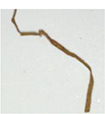	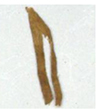	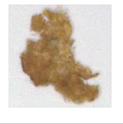	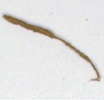	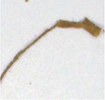	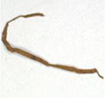

The expression for the cross entropy loss function is


(1) $$C_{l\,o\,s\,s}=-\frac{1}{m}\,\sum_{i=1}^{m}\sum_{j=1}^{j=4}y_{j\,i}\,\text{log}\,\left({\hat{y}}_{j\,i}\right)$$

and the expression for the focal loss function is


(2) $$F_{l\,o\,s\,s}=-\frac{1}{m}\,\sum_{i=1}^{m}{\left(1-p_{i}\right)}^{\gamma }\,\log\left(p_{i}\right)$$

where, *C*_*loss*_, cross-entropy loss function; *F*_*loss*_, focal loss function; *m*, number of the current batch of images input to the network; *y*_*ji*_, authentic label; ${\hat{y}}_{j\,i}$, predicted label; γ, modulation factor; *p_i_*, probability of softmax output.

Equation 2 gives the contribution of the classification samples to the loss. The introduction of the modulation factor γ makes the model weaken the contribution of the loss values of the easily identified samples and gives higher weight to difficult-to-classify samples during training. To void the model value to be equal to 0 or 1 during training, the set value range is [0.005, 0.995], if values below 0.005 are set to 0.005 and values above 0.995 are set to 0.995.

#### Influence of γ on model accuracy

**FIGURE 14:**
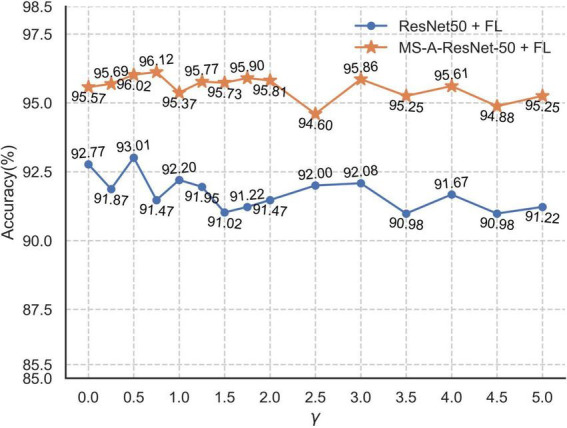
Accuracy rates of ResNet50 + FL and MS-A-ResNet-50 + FL networks under different γ.

The size of the modulation factor γ mainly affects the contribution of the loss value of the sample. With increasing γ, the contribution of the sample loss value is suppressed, with the loss values of easily identified samples suppressed more. Figure 14 shows the accuracy of the ResNet50 and MS-A-ResNet-50 networks on the test set for different γ values.

As shown in Figure 14, the ResNet50 + FL (Focal Loss) network has its highest accuracy (93.01%) when γ = 0.5, and MS-A-ResNet-50 + FL has its highest accuracy (96.12%) when γ = 0.75. The multi-scale structure improves the performance of the model effectively. This study mainly explores the accuracy of the MS-X-ResNet network and selects γ = 0.75 as the modulation factor of the focal loss function.

### Implementation details

#### Test platform

The experiment in this study is based on the Windows 10 operating system; the GPU is GeForce GTX 3080 (10 GB video memory), the processor is Intel(R) Core(TM) i7-12700K CPU at 3.61 GHz, and the running memory is 64 G. Model building, training, and testing are implemented in Python language, based on the PyTorch deep learning framework, the parallel computing framework uses CUDA 11.3 version, and the development environment uses Pycharm.

#### Network evaluation

This study uses accuracy rate (ACC), precision rate (P), recall rate (R), F1 score (F1), and avg_metrics as evaluation indicators (Table 7). In Table 7, *TP, TN, FP, FN* and *k*_*i*_ represent true positive, true negative, false positive, false negative, and the evaluation indicators of samples of a tobacco shred type, respectively. Among the evaluation indicators, F1 is a comprehensive indicator that fuses precision rate and recall rate, and higher F1 values correspond to a better model (Kang and Gwak, 2021).

**TABLE 7:** Table of evaluation indicators.

No.	Evaluation indicators	Calculating formulas
1	Accuracy rate (*ACC*)	$A\,C\,C=\frac{T\,P+T\,N}{T\,P+T\,N+F\,P+F\,N}$
2	Precision rate (P)	$P=\frac{T\,P}{T\,P+F\,P}$
3	Recall rate (R)	$R=\frac{T\,P}{T\,P+F\,N}$
4	F1 score (*F1*)	$F\,1=2\times \frac{P\times R}{P+R}$
5	Average metrics (*avg*_*metrics*)	$a\,v\,g\,\_\,m\,e\,t\,r\,i\,c\,s=\frac{\sum_{i=1}^{i=4}k_{i}}{4}\times 100\%$

We use the four indicators, namely, accuracy rate, precision rate, recall rate, and F1 score, for performance evaluation of our improved model and other contrasting models. Weighted or equally weighted averaging methods can be chosen when calculating the average indicator. Due to the differences in classification difficulty of the different tobacco shred images, the equally weighted average method was chosen in this study.

#### Training details

We use bilinear interpolation (Guo et al., 2011; Kirkland, 2016) to ensure the image quality after scaling as much as possible. Each image in the tobacco shred dataset is scaled to 224 × 224 pixel size. Each channel of data is standardized with a mean of 0.5 and a SD of 0.5. The training set images are shuffled randomly before input to reduce the effect of image order on the model. Through the function of the optimization algorithm, the model performs gradient descent after multiple iterations and attenuates the learning rate during the model training process so that the model can obtain better classification performance.

The optimization algorithm of the model is Adam: the initial learning rate is 10^–4^, the weight decay is 10^–4^, β_1_ = 0.9, β_2_ = 0.99, and ε = 10^–8^. The batch size is set to 32. When training with the focal loss function, the initial learning rate is set to 10^–3^ because the focal loss function reduces the strength and frequency of network updates. The maximum number of iterations is set to 50. After each iteration, the model’s accuracy is tested on the test set, and the model and results generated by each iteration are retained.

## Results

### Multi-Scale-X-ResNet: Comparison with other networks

We chose the following models as baseline models: VGG16 (Rodríguez et al., 2018; Simonyan and Zisserman, 2019), GoogleNetV3 (Szegedy et al., 2016), ResNet50 (He et al., 2016), ResNet101 (Szegedy et al., 2015), and MobileNetV2 (Sandler et al., 2018).

Table 8 shows the performance indices obtained using the cross-entropy function for the baseline networks. It can be seen from Table 8 that the VGG16 network performs the worst among the baseline networks with an accuracy rate of 90.33%, and ResNet50 performs the best with an accuracy rate of 92.77%. The GoogleNetV3 and MobileNetV2 networks achieved 91.43 and 91.83% classification accuracy, respectively. The GoogleNetV3 network’s Inception structure includes the fusion of various scale features, which enables it to achieve excellent performance. The MoblileNetV2 network introduces an Inverted residual block, causing a smaller loss of high-dimensional information after passing through the ReLU activation function. In addition, the linear bottleneck is used to replace the nonlinear activation to prevent the activation function from filtering too much practical information during low-dimensional transformation, thereby improving the classification performance. From the classification performance of the GoogleNetV3 and MobileNetV2 networks, low-dimensional features contain the details of tobacco shred images, so the loss of this information reduces the network’s tobacco shred classification performance.

**TABLE 8:** Performance index of baseline models with tobacco shred images.

Model	Accuracy (%)	Avg_Precision (%)	Avg_Recall (%)	Avg_F1 score (%)
VGG16	90.33	90.33	90.20	90.22
GoogleNetV3	91.43	91.32	91.31	91.30
MobileNetV2	91.83	91.97	91.72	91.72
ResNet50	92.77	92.66	92.67	92.66
ResNet101	91.87	91.79	91.76	91.75

**FIGURE 15:**
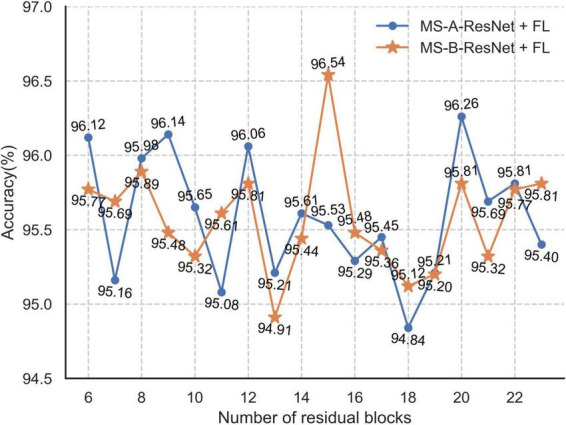
Accuracy with different number of residual blocks in MS-X-ResNet.

Figure 15 shows the test accuracy of MS-X-ResNet using the focal loss function with different numbers of residual blocks. From Figure 15, the accuracy of the MS-A-ResNet + FL network is 94.84–96.26%, and the accuracy of the MS-B-ResNet + FL network is 94.91–96.54%.

Supplementary Table 3 shows the best test accuracies of the MS-X-ResNet network, in which the accuracy of the MS-A-ResNet-92 network using the focal loss function test set is 96.26%, and the accuracy of MS-B-ResNet-77 is 96.54%. The MS-B-ResNet-77 network has advantages in classifying tobacco shred images, so this network is selected to complete the tobacco shred classification task.

The performance of different networks using the focal loss function is given in Table 9, which shows that MS-B-ResNet-77 + FL has the best performance with the highest accuracy, average precision (Avg_Precision), average recall (Avg_Recall), and average F1 score (Avg_F1 Score). Compared with the baseline networks in Table 8, the performance of all the baseline networks has also improved.

**TABLE 9:** Performance comparison of different models using focal loss function.

Model	Accuracy (%)	Avg_Precision (%)	Avg_Recall (%)	Avg_F1 score (%)
VGG16 + FL	90.53	90.40	90.40	90.36
GoogleNetV3 + FL	91.75	91.66	91.65	91.64
MobileNetV2 + FL	93.78	93.72	93.70	93.69
ResNet50 + FL	93.01	92.89	92.91	92.89
ResNet101 + FL	92.04	91.97	91.92	91.90
MS-A-ResNet-92 + FL	96.26	96.24	96.21	96.22
MS-B-ResNet-77 + FL	96.54	96.50	96.50	96.49

We choose the high-performing MobileNetV2 + FL and ResNet50 + FL baseline networks to make a detailed comparison with MS-B-ResNet-77 + FL for each tobacco shred type classification. The performance of each model on the test set is shown in Table 10. The precision of MobileNetV2 + FL (92.55%) for expanded tobacco silk is 3.02% higher than for ResNet50 + FL (89.53%), and the probability of other types of tobacco shred being mistaken for expanded tobacco silk decreases. The recall rate of MobileNetV2 + FL for tobacco silk (91.74%) is 3.97% higher than for the ResNet50 + FL network (87.77%), indicating that MobileNetV2 + FL has dramatically improved the recognition accuracy of tobacco silk. Compared with ResNet50 + FL, MobileNetV2 + FL has slightly better performance, especially in classifying between expanded tobacco silk and tobacco silk, which is the most challenging classification task because these have no apparent macro-scale characteristic differences.

**TABLE 10:** Performance of the three models on the test set of tobacco shred.

Tobacco shred	MoboleNet V2 + FL			ResNet50 + FL			MS-B-ResNet-77 + FL		
			
	Precision (%)	Recall (%)	F1 score (%)	Precision (%)	Recall (%)	F1 score (%)	Precision (%)	Recall (%)	F1 score (%)
Expanded tobacco silk	92.55	88.12	90.28	89.53	88.94	89.23	94.70	94.39	94.54
Cut stem	93.90	95.74	94.81	93.11	95.09	94.09	96.75	97.38	97.06
Tobacco silk	89.52	91.74	90.62	89.85	87.77	88.80	94.85	94.38	94.61
Reconstituted tobacco shred	98.91	99.22	99.06	99.07	99.84	99.45	99.69	99.84	99.76
Average	93.72	93.71	93.69	92.89	92.91	92.89	96.50	96.50	96.49

Compared with ResNet50 + FL, MS-B-ResNet-77 + FL has obviously better performance, especially in classifying between expanded tobacco silk and tobacco silk. The precision of MS-B-ResNet-77 + FL (94.70% for expanded tobacco silk and 94.85% for tobacco silk) is 5.17 and 5% higher than ResNet50 + FL (89.53 and 89.85%, respectively). The recall rate of MS-B-ResNet-77 + FL (94.39% for expanded tobacco silk and 94.38% for tobacco silk) is 5.45 and 6.61% higher than for ResNet50 + FL (88.94 and 87.77%, respectively). The average precision, recall rate, and F1 score (96.50, 96.50, and 96.49%, respectively) of MS-B-ResNet-77 + FL are all higher than for ResNet50 + FL (92.89, 92.91, and 92.89%, respectively).

Overall, MS-B-ResNet-77 + FL performs the best in classification tests of each tobacco shred type compared with MobileNetV2 + FL and ResNet50 + FL. We believe this is because MS-B-ResNet-77 + FL adds a multi-scale structure, retaining detailed information about very small targets in the shallow feature map and reducing feature loss.

### Time complexity

When deep learning is used for tobacco shred image classification tasks and performance indicators such as model precision, recall rate, and F1 score, the time complexity is also important. Too high time complexity will affect the actual deployment and application in the field. To ensure that the model proposed in this study can run smoothly on a computer without GPU, MS-X-ResNet model is compared with the baseline model to emphasize that the modified model can be practically used for tobacco shred image recognition. The test is performed on a computer with an Inter(R) Core(TM) i7-12700K CPU at 3.61 GHz and a running memory of 64 G. Only one image is used per test to test the actual model execution speed, which is most likely to occur in actual use. We calculate the average inference time for 2,458 tobacco shred images in the entire test set, and the results are shown in Supplementary Table 4. It can be found that our proposed MS-B-ResNet-77 network performs on par with ResNet-101. The network can process nine datasets containing a single image in 1 s. At the same time, since replacing the model’s loss function with Focal loss is performed during the model training process, it will not affect the inference execution speed of the model.

## Discussion

In the deep learning image classification task, the dataset’s quality is one of the core factors affecting the model’s classification accuracy. The tobacco shred residue image size can be too small, and differences between the different types of tobacco shred residue images are not apparent. These residue images eventually become dirty data in the sample. Therefore, we first screened the tobacco shred residue. In addition, our dataset and current research are constructed for the subsequent detection of tobacco shred components and actual field production use. The shooting field of view is much larger than the single tobacco shred field of view, so the parameters of the subsequent shooting platform do not need to be changed.

Furthermore, different tobacco shred types have significant differences in thickness, light transmittance, and color depth, resulting in substantial challenges to the binarization of the tobacco shred images. We have performed many tobacco shred image processing experiments. The simple, adaptive, and Otsu’s threshold binarization performance were compared. Finally, a block threshold binarization method was designed, and its parameter settings and performance were researched. After follow-up contour screening, adjustment, and cropping of the ROI area, a dataset for the four tobacco shred types was constructed.

Network selection, structure adjustment, and hyperparameter optimization are complicated in deep learning classification tasks. In this study, the ResNet network’s Stage 3 and Stage 4 features are fused to obtain the MS-ResNet network. From the performance of ResNet50 and ResNet101, it can be concluded that different numbers of Stage 3 blocks affect the model’s performance in tobacco shred image classification tasks. To further improve the model’s performance, the number of blocks in MS-ResNet’s Stage 3 and the impact of the model classification performance are explored, and MS-A-ResNet is obtained. We also reduced the number of Stage 2 blocks in MS-A-ResNet from 4 to 3 to build MS-B-ResNet with reduced complexity. Since the image classification difficulty of different tobacco shred types is quite different, the focal loss function replaced the cross-entropy loss function to accommodate the test characteristics of the tobacco shred samples.

The advantages and disadvantages of the proposed methods and follow-up research directions are now discussed. The current, complete tobacco shred classification scheme has the following benefits:

1. The proposed scheme provides a complete set of solutions for screening samples, taking images, processing images, and building datasets for tobacco shred classification.
2. The number of raw tobacco shred data samples is large, consists of actual digital images, and provides a better generalizability for actual field use.
3. The MS-X-ResNet network demonstrates excellent performance in classifying and recognizing tobacco shreds. It has a good classification capability for tobacco shred images having different sizes and types.
4. The execution speed of the proposed network is fast, so the execution time in identifying tobacco shred images is low, saving computing resources and identification time.
5. The field of view of the shooting platform is much larger than the contour field of view of a single tobacco shred. The current shooting platform parameters and experimental data can be directly used in the subsequent online component detection research of tobacco shreds, providing good continuity with practical scenarios for application.

The proposed scheme also has the following limitations:

1. The specific size information of a tobacco shred image is not used in the model, and the tobacco shred images are scaled to 224 × 224 pixels before being sent to the network. Adding the size characteristics of tobacco shreds may improve the network’s performance.
2. Different convolutional network models have different sensitivities to the focal loss modulation factor γ. Focal loss with the same γ value for all convolutional networks in this study may not achieve optimal performance for each network.

Follow-up work is as follows: (1) the designed black box image acquisition device needs to be optimized further to obtain higher quality images, especially to improve the lighting installation methods to prevent shadows. (2) The block threshold binarization used was traditional and was not robust enough. Semantic segmentation can be used for tobacco shred extraction. (3) The influence of different tobacco shred types’ geometric features, such as length, width, area, and aspect ratio, on the classification can be explored, and these features can be input into the network with the image information. (4) The γ parameter of the focal loss can be optimized for different convolutional network models, and the network performance can be evaluated after obtaining the optimal tuning factor corresponding to each network. (5) This study was performed in the laboratory, and the method must be validated in practical scenarios for application.

## Conclusion

This study describes the construction of an experimental platform and subsequent image processing to establish a tobacco shred dataset aimed at the practical classification and identification of tobacco shred types on a production line. Based on ResNet50, the MS-B-ResNet-77 network is proposed. Performance indicators including accuracy, precision, recall rate, F1 score, and time complexity are used to evaluate the network’s performance. This research achieves the following innovations:

1. A block threshold binarization method is designed by combining the processing ideas of the adaptive and Otsu thresholds. Further, contour screening and ROI area cropping steps are designed to segment tobacco shred images, and complete tobacco shreds can be obtained by expanding the ROI area.
2. The MS-X-ResNet network is proposed by fusing the multi-scale Stage 3 low-dimensional and Stage 4 high-dimensional features to reduce overfitting risk. The number of blocks in each stage is optimized to improve the model’s classification performance with lower complexity. Furthermore, performance evaluation for MS-X-ResNet is performed, and it is compared with multiple convolutional network models for tobacco shred image classification and recognition.
3. The focal loss function is applied to tobacco shred classification, which alleviates the influence of varying degrees of classification difficulty for different tobacco shred types on the model. This function effectively improves the accuracy and stability of the model.

## Data availability statement

The datasets presented in this article are not readily available because the data analyzed in this study is subject to the following licenses/restrictions: our data is protected by copyright. For data sources, contact the Institute of Zhengzhou Tobacco Research Institute of CNTC, website at: https://www.ztri.com.cn/. Requests to access the datasets should be directed to WZ.

## Author contributions

QN and JL conceived the idea, designed the experiments, collected and analyzed data, and wrote the manuscript. JL and QY selected and trained algorithms. YJ and XC provided experimental equipment, participated in data collection, and tested the method. WZ provided the original tobacco shred samples. WZ and QY determined the platform construction program. All authors contributed to the article and approved the submitted version.
