# Supplementary material for: Tobacco shred varieties classification using Multi-Scale-X-ResNet network and machine vision
Source: Front Plant Sci. 2022 Aug 18;13:962664. doi: 10.3389/fpls.2022.962664 (PMC9433752; doi:10.3389/fpls.2022.962664)
Supplement: Supplementary file 1 [file Data_Sheet_1.docx]

Supplementary Material

Supplementary Tables 1 | Otsu's threshold Binarization performance evaluation table.

| **Binarization method** | **Complete samples/Total samples** | **The proportion of complete samples** | **Excution Time(s)** |
| --- | --- | --- | --- |
| Otsu’s threshold | 6896/8202 | 84.08% | 1544.65 |

Supplementary Tables 2 | Number of blocks of different ResNet.

| **Model** | **d_1_:d_2_:d_3_:d_4_:** |
| --- | --- |
| ResNet50 | 3:4:6:3 |
| ResNet101 | 3:4:23:3 |
| A-ResNet | 3:4:N:3 |
| B-ResNet | 3:3:N:3 |

Supplementary Tables 3 | Best test accuracy of ResNet and MS-X-ResNet.

| **Model** | **d1:d2:d3:d4:** | **Accuracy** |
| --- | --- | --- |
| MS-A-ResNet-92 + FL | 3:4:20:3 | 96.26% |
| **MS-B-ResNet-77 + FL** | **3:3:15:3** | **96.54%** |

Supplementary Tables 4 | Execution time for the networks.

| **Model** | **Excution Time(miliseconds)** |
| --- | --- |
| GoogleNetV3 | 39 |
| VGG16 | 78 |
| MobileNetV2 | 23 |
| ResNet50 | 53 |
| ResNet101 | 93 |
| MS-A-ResNet-92 | 246 |
| MS-B-ResNet-77 | 103 |
